# 1H-NMR-Based Metabolic Analysis of Human Serum Reveals Novel Markers of Myocardial Energy Expenditure in Heart Failure Patients

**DOI:** 10.1371/journal.pone.0088102

**Published:** 2014-02-05

**Authors:** Zhiyong Du, Anna Shen, Yuli Huang, Liang Su, Wenyan Lai, Peng Wang, Zhibing Xie, Zhiquan Xie, Qingchun Zeng, Hao Ren, Dingli Xu

**Affiliations:** 1 Department of Cardiology, Nanfang Hospital, Southern Medical University, Guangzhou, China; 2 Key Laboratory For Organ Failure Research, Ministry of Education of the People's Republic of China, Guangzhou, China; 3 Department of Cardiology, The Third Hospital of Southern Medical University, Guangzhou, China; 4 Department of Cardiology, Guangzhou General Hospital of PLA, Guangzhou, China; 5 Department of Rheumatology, Nanfang Hospital, Southern Medical University, Guangzhou, China; Mayo Clinic, United States of America

## Abstract

**Objective:**

Elevated myocardial energy expenditure (MEE) is related with reduced left ventricular ejection fraction, and has also been documented as an independent predictor of cardiovascular mortality. However, the serum small-molecule metabolite profiles and pathophysiological mechanisms of elevated MEE in heart failure (HF) are still lacking. Herein, we used 1H-NMR-based metabolomics analysis to screen for potential biomarkers of MEE in HF.

**Methods:**

A total of 61 subjects were enrolled, including 46 patients with heart failure and 15 age-matched controls. Venous serum samples were collected from subjects after an 8-hour fast. An INOVA 600 MHz nuclear magnetic resonance spectrometer with Carr-Purcell-Melboom-Gill (CPMG) pulse sequence was employed for the metabolomics analysis and MEE was calculated using colored Doppler echocardiography. Metabolomics data were processed using orthogonal signal correction and regression analysis was performed using the partial least squares method.

**Results:**

The mean MEE levels of HF patients and controls were 139.61±58.18 cal/min and 61.09±23.54 cal/min, respectively. Serum metabolomics varied with MEE changed, and 3-hydroxybutyrate, acetone and succinate were significantly elevated with the increasing MEE. Importantly, these three metabolites were independent of administration of angiotensin converting enzyme inhibitor, β-receptor blockers, diuretics and statins (*P*>0.05).

**Conclusions:**

These results suggested that in patients with heart failure, MEE elevation was associated with significant changes in serum metabolomics profiles, especially the concentration of 3-hydroxybutyrate, acetone and succinate. These compounds could be used as potential serum biomarkers to study myocardial energy mechanism in HF patients.

## Introduction

Cardiac energy and metabolism are tightly regulated for a high and constant workload, however such regulation becomes compromised in heart failure [1], [2], [3]. It had been documented that elevated myocardial energy expenditure (MEE) is related with left ventricular ejection fraction (LVEF), as well as an independent predictor of cardiovascular mortality [4]. For this reason, significant efforts have been directed towards the pathophysiological mechanisms of elevated MEE. The conventional way to estimate MEE in the failing heart is to calculate the amount of O_2_ extracted by the left ventricle from arterial blood. However, this method is difficult and invasive. Some advanced imaging techniques, such as positron emission tomography, single-photon emission tomography and phosphorus-31 magnetic resonance (^31^P-MR) have allowed the non-invasive measurement of cardiac metabolism [5], [6]. Doppler echocardiography has been employed to estimate MEE by integrating a number of physiological factors contributing to myocardial energetic requirement in addition to wall stress, i.e., stroke volume and left ventricle (LV) ejection time [7].

Metabolomic analysis, the systematic study of small-molecule metabolite profiles, has been used to identify potential biomarkers that provide new insights into biological processes [8]. Recently, studies have shown that there are significant metabolic differences in the serum [9], [10] and urine [11] samples between heart failure (HF) patients and the control subjects. This finding suggests that concentration of some serum metabolites may well correlate with MEE levels, the former of which could be conveniently monitored by metabolomic analysis methods such as ^1^H NMR spectroscopy. However, a metabolomic investigation of serum metabolites associated with different MEE levels in HF patients has not yet been reported. In the present study, we performed NMR-based metabolomic analysis on serum obtained from HF patients, in an effort to determine the serum metabolites differences of patients with different MEE levels.

## Materials and Methods

### Ethics statement

This study was carried out in accordance with the Helsinki Declaration. This study was approved by the institutional ethics committee of Nanfang Hospital. All subjects had provided their written informed consent to participate in this study.

### Subjects

For this study, 46 HF patients (Male: 35; Female: 11, aged 28–87 years, mean age 62.7±13.0 years) from the cardiology department of Nanfang Hospital were recruited. All patients were staged in NYHA (New York Heart Association) class II (n = 11), III (n = 18) and IV (n = 17). The exclusion criteria were: (1) type 2 diabetes mellitus and other metabolic disease; (2) acute or chronic inflammatory conditions; (3) malignancies, and (4) significant respiratory pathology. Fifteen age-matched controls with normal cardiac function (Male: 8; Female: 7, aged 30–80 years, mean age 57.8±10.8) were recruited from the health management center in Nanfang Hospital.

### MEE measurement

MEE was measured with a Siemens Sequoia 512 Encompass ultrasound system, using the method described previously [4], [7], which assumed that (1) end-systolic stress is a representative measure of the systolic tension applied to the myocardium during the ejection phase, (2) Doppler echocardiography was permitted to estimate the mass moved by the myocardium, and (3) trans-aortic Doppler flow could be used to measure the period during LV ejection (LV ejection time, LVET). Finally, MEE was calculated as:

MEE (kcal/min)  = LV circumferential end-systolic wall stress (cESS) ×LVET×LV stroke volume (LVSV)×HR×4.2×10^−4^.

### Sample collection

Venous blood was collected from the antecubital vein on the same day of MEE measurement, coagulated at room temperature for 10 min, and centrifuged at 3000 g at 4°C for 10 min. The resulting supernatant serum was collected and stored at −80°C.

### Sample preparation and ^1^H-NMR spectroscopy

Stored serum samples were thawed prior to NMR analysis. 300 µl of serum was mixed with 200 µl D_2_O and 100 µl 3-trimethylsilyl-^2^H_4_-propionic acid sodium salt (TSP) in D_2_O (1 mg/ml). After centrifuged at 13000 g for 10 min, 550 µl aliquots of the supernatant were transferred into 5-mm NMR tubes for analysis.

^1^H-NMR spectra of the serum samples were acquired on a Varian INOVA 600 MHz spectrometer at 27°C using Carr-Purell-Meiboom-Gill (CPMG) spin-echo pulse sequence with a total spin-spin relaxation delay (2 nτ) of 320 ms. The free induction decays (FIDs) were collected into 32 k data points with a spectral width of 8000 Hz and 64 scans. The FIDs were zero-filled to double size and multiplied by an exponential line-broadening factor of 0.5 Hz prior to Fourier transformation (FT). All serum ^1^H-NMR spectra were manually phased and baseline corrected using VNMR 6.1C software (Varian Inc., Palo Alto, CA, USA). Each spectrum over the range of δ 0.4–4.4 was data-reduced into integrated regions of equal width (0.01 ppm). The regions containing the resonance from residual water (δ4.6–5.1) were removed. The integral values of each spectrum were normalized to a constant sum of all integrals in a spectrum. Identification of metabolites in spectra was accomplished based on information in the literature and the Chenomx NMR Suite 5.0 (Chenomx, Calgary, Canada).

### Statistical analysis

Clinical, functional data and metabolite concentration are presented as mean ± SD or percentages as appropriate. All the included subjects were divided into three quartile groups according to MEE level. Differences between MEE groups were evaluated using the χ^2^ test for discrete clinical variables and by one-way ANOVA for continuous variables. Differences within groups were evaluated using the Bonferroni method if variables were equal; otherwise Dunnett's T3 test was used. The resulting integral NMR data were imported into SIMCA-P (version 11.5; Umetrics, Umeå, Sweden) for multivariate analysis. The CPMG data were mean-centered and Pareto-scaled prior to analysis. Principle component analysis was performed to observe the samples distribution. To remove the variations not correlated to the group membership, the data were preprocessed using orthogonal signal correction (OSC) followed by PLS analysis.

## Results

### Baseline characteristics of variable MEE groups

The mean ejection fraction in HF patients was 39.59±10.94%, 48% showed mildly reduced EF (40%–54%), and 52% had severely reduced EF (≤40%). Medical treatment for the HF patients consisted of diuretics (71.7%), β-blockers (73.9%), angiotensin converting enzyme inhibitors or angiotensin II receptor blocker (69.6%), and statins (28.3%). The mean MEE levels of HF patients and controls were 139.61±58.18 cal/min and 61.09±23.54 cal/min, respectively. All the included subjects (46 HF patients and 15 controls) were divided into three quartile groups according to MEE level. Those with an MEE<72.53 cal/min were included in the low MEE group (Q1); 72.53≤MEE<176.75 cal/min in the intermediate MEE group (Q2+Q3); MEE≥176.75 cal/min in the high MEE group (Q4). Distribution of participants and baseline characteristics of the three groups are shown in Table 1. Participants in the high MEE group had higher NYHA classification than other groups (χ^2^ = 33.02, *P*<0.001). Those in the high MEE group had higher fasting glucose, very low density lipoprotein (VLDL), creatinine, uric acid, and BNP levels and a lower LVEF than those in the low MEE group.

**Table 1:** Demographic and clinical characteristics of patients in different-MEE groups.

	Low MEE	Intermediate MEE	High MEE	*P* value
	(n = 15)	(n = 31)	(n = 15)	
Participants' distribution				<0.001
Healthy	10(66.7%)	5(16.1%)	0	
NYHA II	2(13.3%)	9(29.0%)	0	
NYHA III	2(13.3%)	6(19.4%)	10(66.7%)	
NYHA IV	1(6.7%)	11(35.5%)	5(33.3%)	
Age (years)	58.67±10.59	61.29±13.57	64.93±12.56	NS
Sex (Male/Female)	8/7	23/8	12/3	NS
BMI (kg/m^2^)	23.23±2.46	23.48±2.94	23.19±2.62	NS
Fasting glucose (mg/dl)	85.41±18.92	85.41±11.89	96.95±12.43^Δ§^	<0.05
Triglyceride (mg/dl)	167.35±123.96	127.50±62.87	116.88±46.93	NS
Total cholesterol (mg/dl)	181.36±56.46	155.07±46.40	167.05±42.54	NS
HDL-cholesterol (mg/dl)	43.70±13.92	39.83±15.47	37.12±11.60	NS
LDL-cholesterol (mg/dl)	87.79±27.84	91.26±34.03	100.16±38.67	NS
VLDL-cholesterol (mg/dl)	48.72±24.75	32.49±16.63	34.80±13.15Δ	<0.05
Creatinine (mg/dl)	0.87±0.34	1.15±0.36Δ	1.38±0.56Δ	<0.01
eGFR (ml/min·1.73 m^2^)	60.36±18.71	55.40±16.48	52.05±22.91	NS
Uric acid (mg/dl)	6.07±2.48	8.30±2.47Δ	9.39±2.76Δ	<0.01
NT-proBNP (pg/ml)	152±2143	2265±3177	4773±5255	----
Log NT-proBNP	2.32±0.71	3.22±0.70Δ	3.59±0.49Δ	<0.01
CRP (mg/dl)	0.55±0.63	0.96±0.75	1.73±1.13^Δ§^	<0.01
LVEF (%)	58±12	46±14	32±6	<0.01
ACEI/ARB	3(20%)	17(54.8%)	12(80%)	<0.05
β-Blockers	3(20%)	18(58.1%)	13(86.7%)	<0.05
Diuretics	3(20%)	17(54.8%)	13(86.7%)	<0.05
Statins	0	8(25.8%)	5(33.3%)	0.057

Δ *vs* low MEE *P*<0.05, ^§^ *vs* intermediate MEE *P*<0.05.

MEE: myocardial energy expenditure; BMI: body mass index; HDL: high densitv lipoprotein; LDL: low-density lipoprotein; VLDL: very low density lipoprotein; eGFR: estimated glomerular filtration rate; NT-proBNP: N-terminal pro brain natriuretic peptide; CRP: c-reactive protein; LVEF: left ventricular ejection fraction; ACEI: angiotensin converting enzyme inhibitor; ARB: angiotensin II receptor blocker.

### ^1^H-NMR spectroscopy of serum from different MEE groups

**Figure 1:**
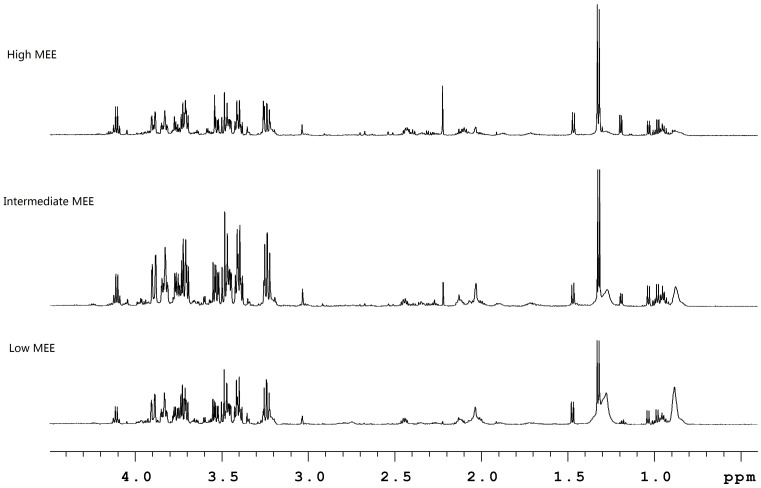
Representative 600^1^H-NMR spectra of serum from low, intermediate and high MEE groups, showing the structural complexity produced by multiple metabolite signals.

Representative standard 1D 600 MHz ^1^H-NMR spectra of serum from low, intermediate and high MEE groups groups showed a large numbers of NMR signals, which indicated the complexity of spectral information obtained from the patient donors (Fig. 1). Identification of metabolites in spectra was accomplished based on information in the literature and the 600 MHz library of the Chenomx NMR Suite 5.0 (Chenomx, Calgary, Canada). As noted in Fig. 1, major metabolites identified in serum included amino acids (valine, alanine, glutamine, methionine, glycine), organic acid (3-hydroxybutyrate, lactate, acetone, succinate, creatine), low-density lipoprotein (LDL) and VLDL, glucose, choline and phosphocholine, N-Acetyl glycoprotein, and trimethylamine-N-oxide.

### Metabolomics analysis of serum from variable MEE groups

**Figure 2:**
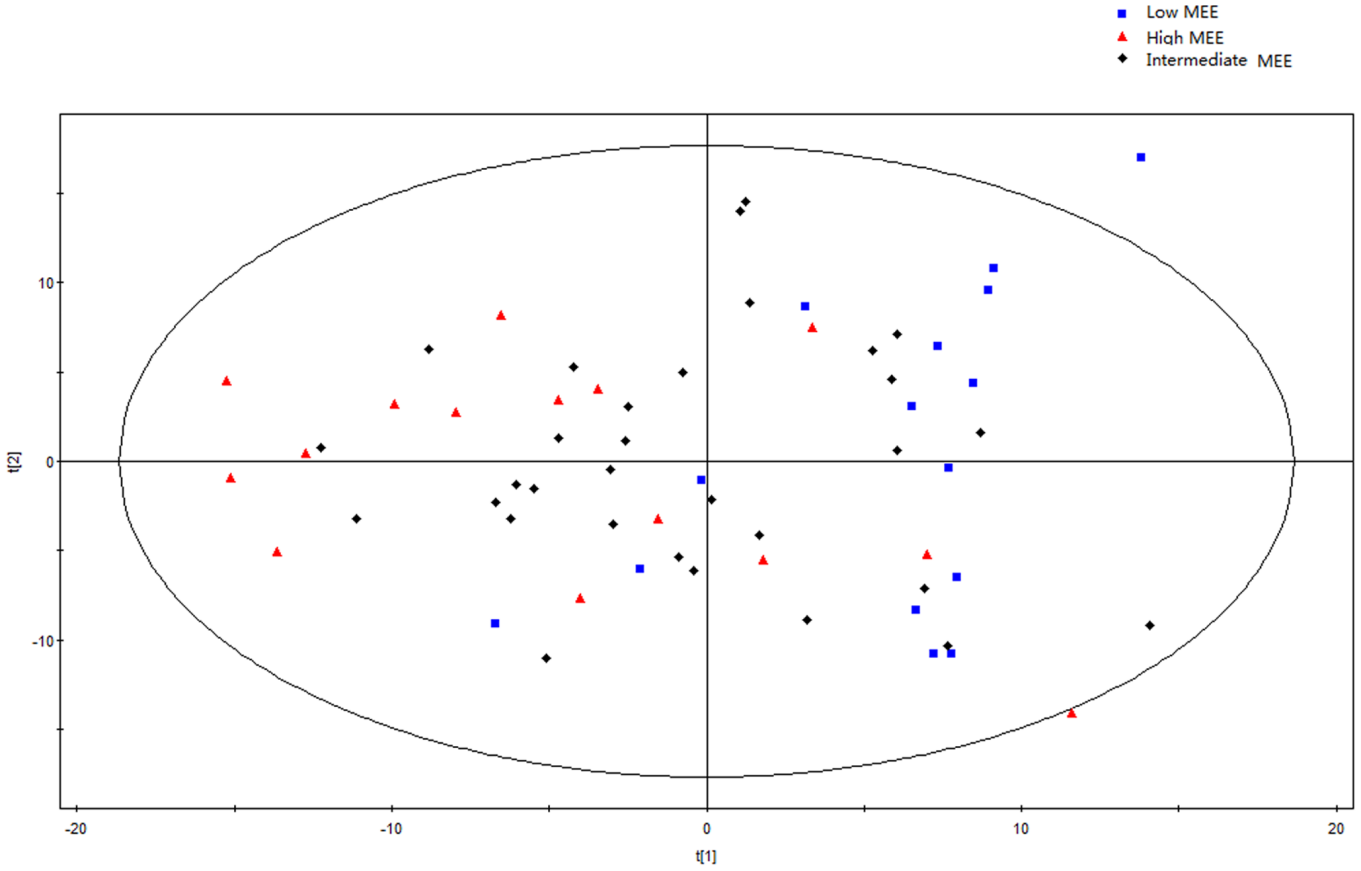
Principal component analysis (PCA) scores plot from low, intermediate and high MEE groups.

**Figure 3:**
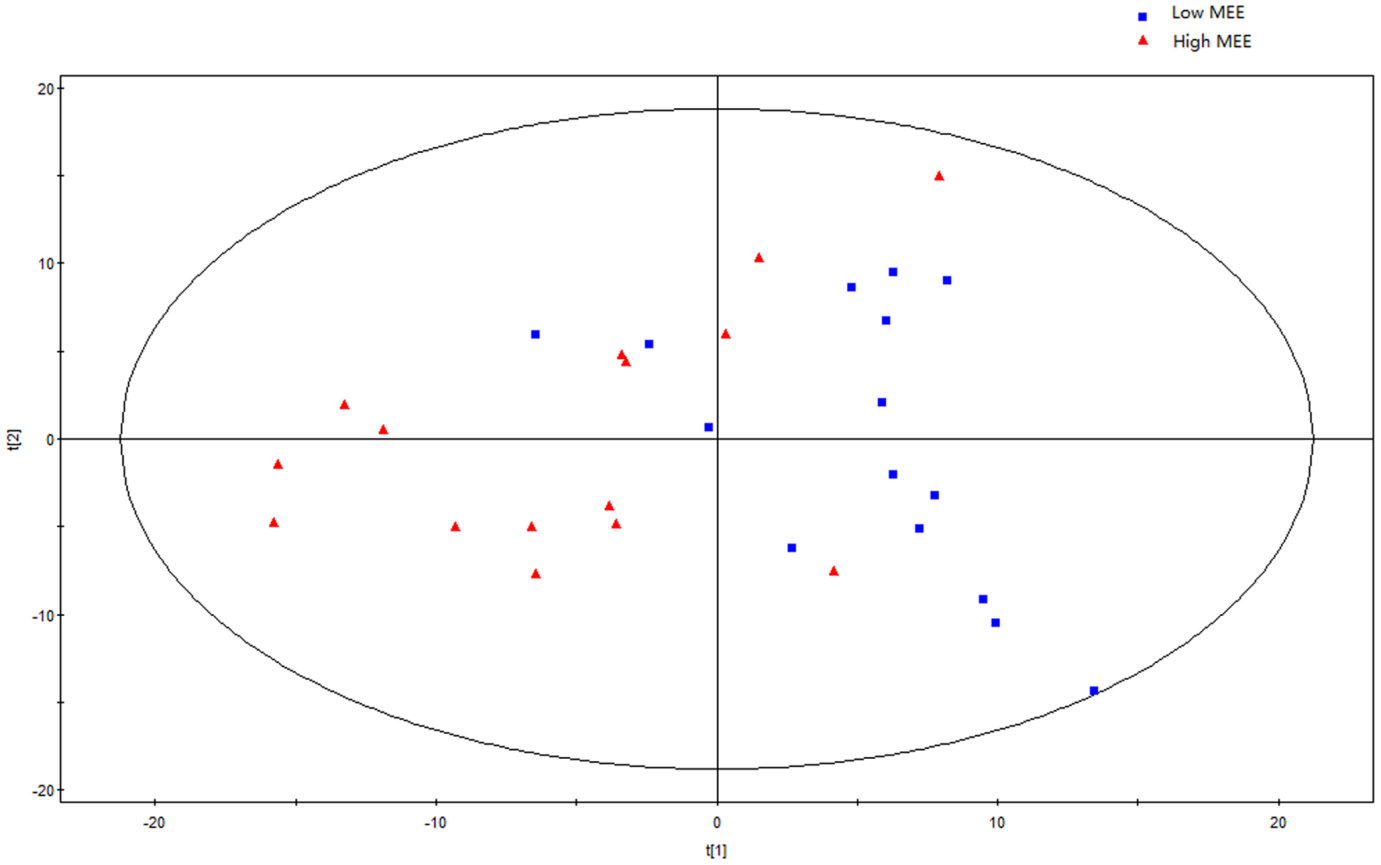
Principal component analysis (PCA) score plot between the low and high MEE groups.

**Figure 4:**
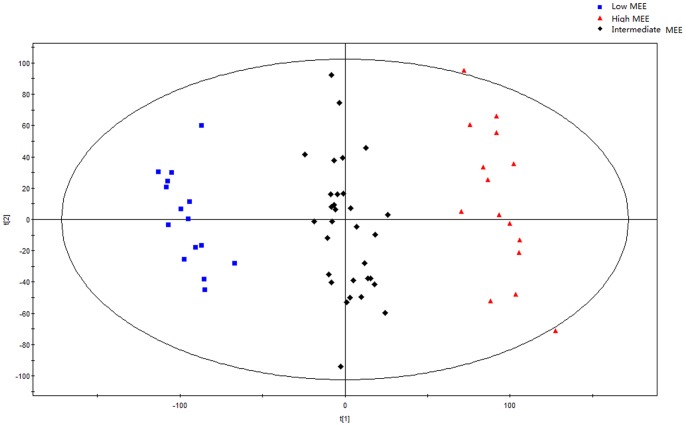
First two components of the OSC-PLS model scores for serum data of low, intermediate and high MEE groups.

**Figure 5:**
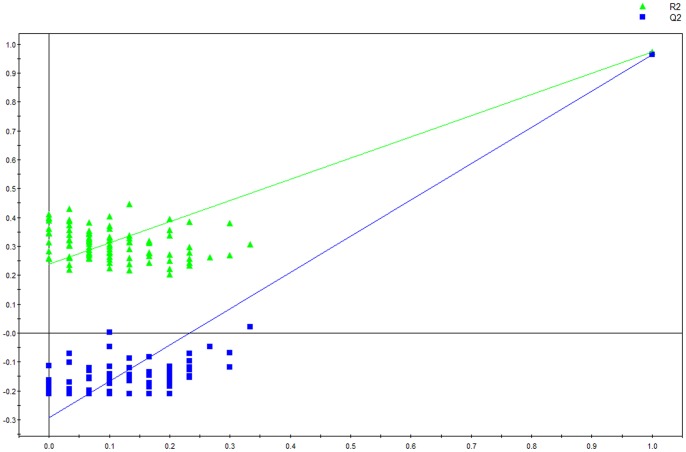
OSC-PLS model validation plot of low, intermediate and high MEE groups.

**Figure 6:**
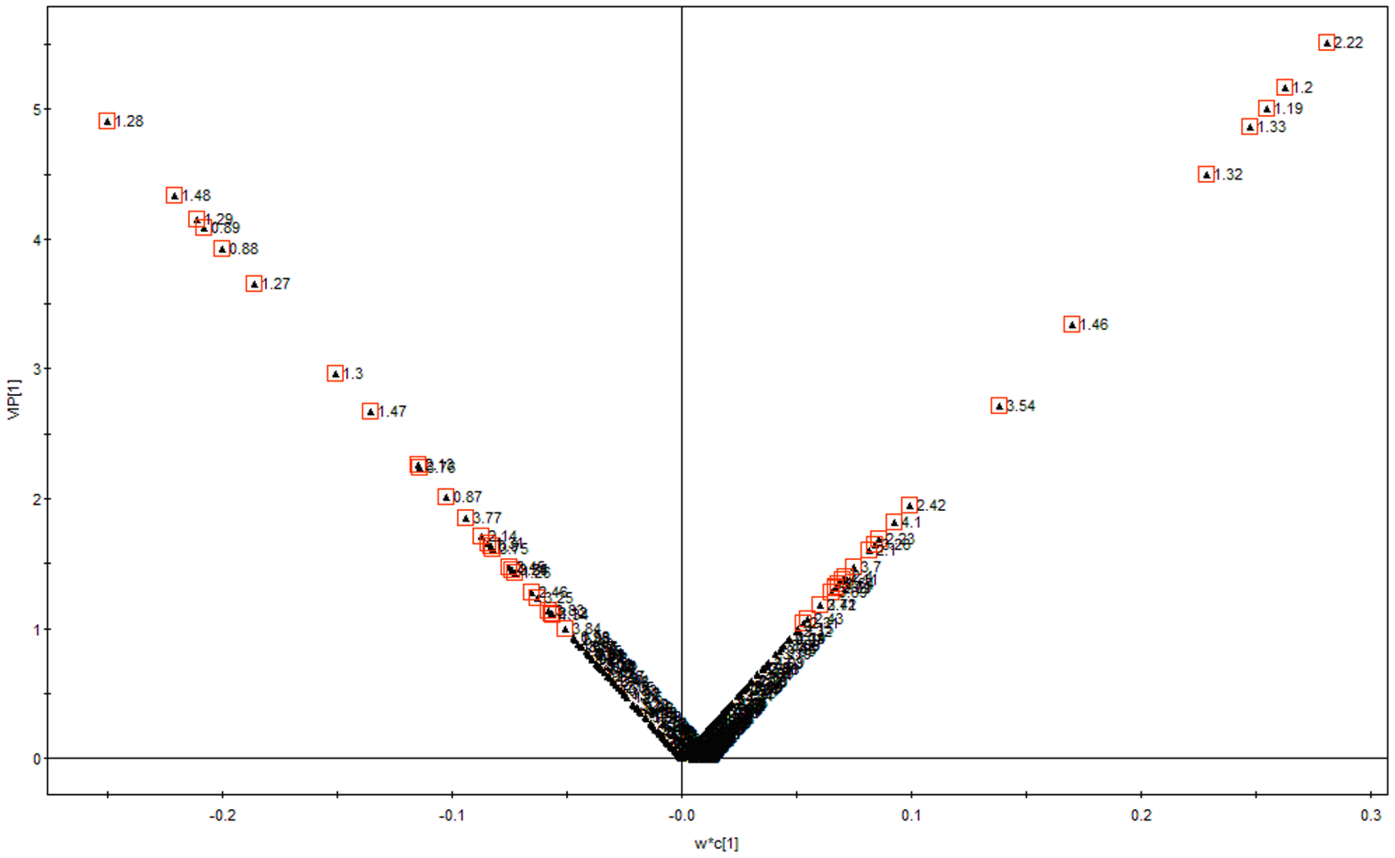
OPLS model V plot of low, intermediate and high MEE groups.

Initially, principal components analysis was applied to examine the intrinsic variation in the serum of all MEE level groups. As shown in Fig. 2, a slight discrimination was observed between all MEE groups by the scores plots of PC1 versus PC2 (R^2^ = 0.694, Q^2^ = 0.347). Pairwise PCA score plots showed a significant difference between the high and low MEE groups by the score plots of PC1 versus PC2 (R^2^ = 0.649, Q^2^ = 0.091) (Fig. 3), but no separation among the low MEE, high MEE and intermediate MEE in serum, respectively. PLS was used to find differential metabolites between groups. Two-dimensional scores plots are shown in Figure S1–S3. Next, the data were preprocessed using orthogonal signal correction (OSC) to remove the variations not correlated to the group membership and to further improve the separation among the three groups[12], [13]. OSC-PLS score plots are shown in Fig. 4; all three groups were located in different clusters (R^2^Y = 92.6% and Q^2^Y = 90.2%). OSC-PLS cross validating parameter are shown in Table 2. Pairwise OSC-PLS also showed discrimination between the groups. Subsequently, this OSC-PLS model was validated with the permutation testing, when Y was randomly permuted 100 times. By comparing R^2^Y and Q^2^Y values of the original model with those of the re-ordered models this type of permutation gauges the statistical significance of the predicting power of the models. It is known that models with a R^2^Y intercept less than 0.3 and a Q^2^Y intercept less than 0.05 indicate valid models [14]. In our study, the permutation test plot showed a proper R^2^Y (∼0.24) and Q^2^Y (−0.29) intercept values, which suggested the validity of our OSC-PLS model (Fig. 5). In the SIMCA-P software, the contribution size of variables in the OSC-PLS model was usually expressed as the value of variable importance in projection (VIP). VIP values and correlation coefficients were shown in the V-plot (Fig. 6). Variables in both terminals of “V” represented a high contribution to size and reliability. Variables were screened according to the VIP value (>1) and 49 potential differential variables were selected. Finally, a total of 18 metabolites were identified according to literature [15], [16] and the HMDB database (Table 3).

**Table 2:** OSC-PLS cross validating parameter between different-MEE groups.

	Numbers	R^2^X	R^2^Y	Q^2^Y
3 Groups	2	0.386	0.926	0.902
Low MEE vs Intermediate MEE	2	0.201	0.930	0.824
Low MEE vs High MEE	3	0.375	0.994	0.927
Intermediate MEE vs High MEE	2	0.195	0.942	0.767

**Table 3:** Metabolites Difference of patients in different-MEE groups.

No	Metabolite	Selected Chemical shift	Group
1	LDL	0.87(br),1.26–1.29(br)	CH_3_-(CH_2_)n-
2	VLDL	0.88–0.90(br),1.30–1.31(br)	CH_3_-(CH_2_)n-
3	Valine	0.98–0.99(d)	γ-CH_3_
4	3-Hydroxybutyrate	1.19–1.20(d)	-CH_3_
5	Lactate	1.32–1.34(d)	-CH_3_
6	Alanine	1.46–1.48(d)	-CH_3_
7	N-Acetyl glycoprotein	2.04(s)	
8	Glutamine	2.09(m)	β-CH_2_
		4.29(t)	α-CH
		2.29(m)	γ-CH_2_
9	Methionine	2.14(s)	S-CH_3_
10	Acetone	2.22–2.23(s)	-CH_3_
11	Succinate	2.42(s)	-CH_2_
12	Creatine	3.03(s)	-CH_3_
13	Choline	3.21(s)	-CH_3_
14	Phosphocholine	3.22(s)	-CH_3_
15	β-Glucose	3.24–3.25(dd)	C-H2
		3.40–3.41(t)	C-H4
		3.45–3.46(m)	C-H5
		3.47–3.48(t)	C-H3
		3.71–3.72(m),3.88–3.90(dd)	C-H6
16	α-Glucose	3.55(s)	C-H2
		3.69–3.70(t)	C-H3
		3.73–3.75(m)	C-H6
		3.83(m)	C-H5&6
17	Trimethylamine-N-oxide	3.26(s)	-CH_3_
18	Glycine	3.97(s)	-CH_2_

S: singlet; d: doublet; t: triplet; q: quartet; m: multiplet; br: broad peak.

**Figure 7:**
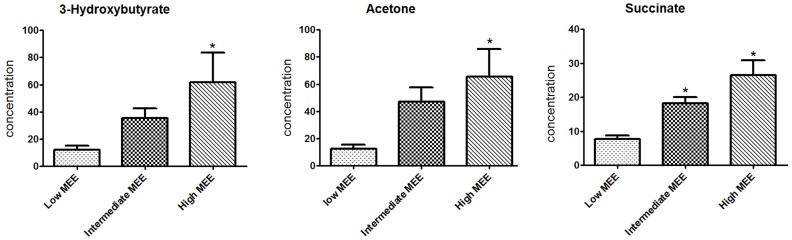
3-hydroxybutyrate, acetone and succinate level in the three MEE groups. * p<0.05 vs. Low MEE.

**Figure 8:**
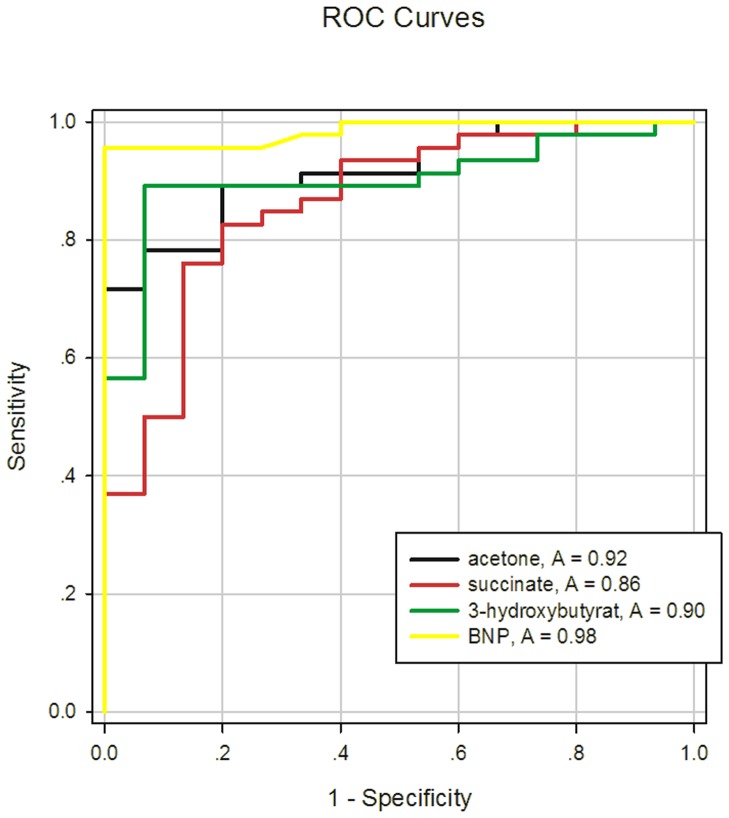
ROC of acetone, 3-hydroxybutyrate, succinate and BNP for CHF.

To validate whether the identified metabolites varied with different MEE levels, metabolites were compared among groups using the one-way ANOVA method (Table 4). It was found that concentration of serum 3-Hydroxybutyrate, acetone and succinate were increased with the increasing of MEE (Table 3, Fig. 7). Importantly, these three metabolites were independent of administration of angiotensin converting enzyme inhibitor, β-receptor blockers, diuretics and statins (*P*>0.05). In addition, the differences of LDL, VLDL, lactate, N-acetyl glycoprotein, choline, phosphocholine, α-glucose, β-glucose, trimethylamine-N-oxide and glycine were not significant among the three groups. Taking the diagnostic criteria of the Framingham Heart Study (FHS) as the benchmark, receiver operating curves (ROCs) of acetone, 3-hydroxybutyrate, succinate and BNP for diagnosis of HF were compared. These results indicated areas under the curve (AUC) were 0.92, 0.90, 0.86 and 0.98, respectively (Fig. 8).

**Table 4:** Different metabolite levels of patients in different-MEE groups.

Metabolite	Low MEE	Intermediate MEE	High MEE	*P*-value
	(n = 15)	(n = 31)	(n = 15)	
LDL	136.4±89.4	93.4±46.8	89.1±63.6	NS
VLDL	119.9±78.8	85.0±43.7	80.6±51.0	NS
Valine	56.8±10.5	48.4±11.8#	47.9±9.3	0.036
3-Hydroxybutyrate	12.1±11.3	35.5±38.6	61.7±84.6#	0.032
Lactate	436.0±143.7	481.1±171.6	483.0±113.1	NS
Alanine	102.1±18.9	82.9±29.0	60.5±28.8^#†^	-<0.001
N-Acetyl glycoprotein	81.7±19.2	79.0±12.3	73.0±10.8	NS
Glutamine	91.9±25.3	73.3±18.3#	70.4±15.8#	0.006
Methionine	55.8±13.5	43.9±15.2#	34.1±15.7#	0.001
Acetone	12.6±11.2	47.0±59.3	65.7±78.5#	0.044
Succinate	7.7±4.2	18.2±9.9#	26.4±17.3#	-<0.001
Creatine	17.1±5.9	23.2±10.3#	18.0±5.0	0.036
Choline	52.8±16.9	45.4±10.2	44.6±10.4	NS
Phosphocholine	101.2±20.0	98.7±21.8	97.2±19.8	NS
β-Glucose	241.9±50.1	245.0±43.6	254.5±39.7	NS
α-Glucose	95.1±16.2	87.1±20.0	79.6±15.4	NS
Trimethylamine-N-oxide	111.8±16.5	125.0±43.5	127.7±40.6	NS
Glycine	83.6±22.8	90.4±36.3	69.6±37.7	NS

# *vs.* low MEE, *P*<0.05; ^†^ *vs* media MEE, *P*<0.05.

## Discussion

Cardiac energy metabolism is strongly related to oxygen supply [17], [18]. MEE has been one of the major indices of myocardial energy metabolism. This study found significant differences in serum metabolic groups between HF patients with different MEE, and screening of these metabolites identified three important serum markers that reflect the myocardial energy metabolism. Our findings thus increase the knowledge of human myocardial metabolism changes in heart failure.

MEE derived from standard echocardiographic measurements is an effective indicator for myocardial bioenergetics and significantly correlated with cardiac function in chronic HF patients, particularly in chronic HF patients with reduced LVEF [4], [19], [20]. Elevated level of MEE and lower adipose mass had been reported to be more effectively to predict cardiac death than EF [4]. In a previous study, our group also showed that in patients with HF after acute myocardial infarction, 12-month treatment with higher doses perindopril can improved myocardial remodeling and left ventricular systolic function, and decreased MEE [21]. These data showed that MEE is important for prognosis of patients with HF.

In the current study, 61 subjects were divided into three groups based on MEE levels. Serum metabolism profiles of these three groups were analyzed via model-identifying methods such as PCA and OSC-PLS. It was observed that OSC-PLS could effectively distinguish the features in different MEE groups, especially for high- and low-MEE groups. Moreover, according to the VIP value and one-way ANOVA results, three metabolites (3-hydroxybutyrate, acetone and succinate) were shown to rise with increasing MEE. Furthermore, the association of these three metabolites with MEE was independent of all treatment regimes including angiotensin converting enzyme inhibitor, β-receptor blockers, diuretics and statins. Both 3-hydroxybutyrate and acetone are ketone bodies, and mainly synthesized from oxidation of fatty acid catalyzed by acetylcoenzyme A in the liver. In general, myocardial energy acquisition via ketones oxidation pathways is dependent on the concentration. When the ketone bodies level is very low in the serum, they only provide a very small part of the energy required by the myocardium. However, in hungry or diabetes mellitus patients, blood ketone bodies level are greatly increased due to low insulin and high fatty acid levels, and they becomes the major energy supplier of the myocardium [22], [23]. It has been found by Lommi *et al* [24], [25]. that ketone bodies levels were significantly higher in patients with chronic HF than the normal population, possibly due to neuroendocrine hormone activation. In this study, we found that 3-hydroxybutyrate and acetone levels were significant increased in HF patients, which is consistent with the above results. More importantly, we found that these ketone bodies level were significantly associated with increasing myocardial energy consumption. Some studies have reported that oxidation and utilization of ketone bodies could inhibit the oxidation of fatty acids by the myocardium [26], [27]. Hence, increase of ketone bodies can promote the conversion of myocardial energy consumption from adult myocardium substrate (i.e., fatty acids) to the embryonal myocardium substrate in HF. Although the mechanism of ketone bodies inhibiting fatty acid metabolism remains unclear, it is inferred to correlate with increasing NADH/NAD+ ratio in the mitochondria, following ketone body utilization to inhibit β-oxidation of fatty acids [28], [29]. Taken these data together, the change of ketone bodies levels may play an important role in pathophysiological mechanisms of elevated MEE in HF.

Succinate is not only an important intermediate metabolite in the tricarboxylic acid cycle but also an important component of the electron respiratory chain complex II. In our study, the succinate level in the high-MEE group was increased by 1.4-fold compared with the intermediate-MEE group, which was a further 2.4-fold higher than the low-MEE group, suggesting that succinate is closely related to myocardial energy metabolism. Pisarenko *et al* [30]. observed that ATP and phosphocreatine levels significantly decreased in the isolated heart, and succinate level was significantly increased in the myocardial perfusate early following reperfusion of the Guinea pig model. Furthermore, succinate also decreased to a normal level together with ATP and phosphocreatine after 30 min. To summarize, these results suggest that succinate level is a marker of myocardial energy. Succinate dehydrogenase is not only the sole enzyme binding mitochondrial inner membrane but also the key enzyme in the tricarboxylic acid cycle, responsible for catalysis from succinic acid to fumaric acid. So the lack of succinic dehydrogenase can cause dilated cardiomyopathy and even HF [31]. It is refered that decreasing activity or suppressed expression of succinate dehydrogenase may be one of the mechanisms for energy production disorder in HF, and as a result, may also induce accumulation of upstream intermediate metabolite succinate, causing elevation of succinate level in the peripheral blood.

### Conclusion

Significant differences were found among serum metabolic groups of different MEE in HF patients, and three serum markers (3-hydroxybutyrate, acetone and succinate) were identified to reflect the myocardial energy metabolism in elevated MEE by using 1H NMR-based metabolic analysis. More advanced technique, such as utility of both 1D noesy and cpmg spectra, may be carried out in future studies to reaffirm the identified metabolites. If these results are confirmed, more studies are needed to investigate the pathophysiological mechanism that dictates elevated levels of these metabolites with increasing MEE in chronic HF.

## Supporting Information

Figure S1 PLS score plot of three MEE groups.
(DOC)

Figure S2 PLS score plot of low MEE and intermediate MEE.
(DOC)

Figure S3 PLS score plot of low MEE and high MEE.
(DOC)
