# Supplementary figures and images for: 1H-NMR-Based Metabolic Analysis of Human Serum Reveals Novel Markers of Myocardial Energy Expenditure in Heart Failure Patients
Source: PLoS One. 2014 Feb 5;9(2):e88102. doi: 10.1371/journal.pone.0088102 (PMC3914925; doi:10.1371/journal.pone.0088102)

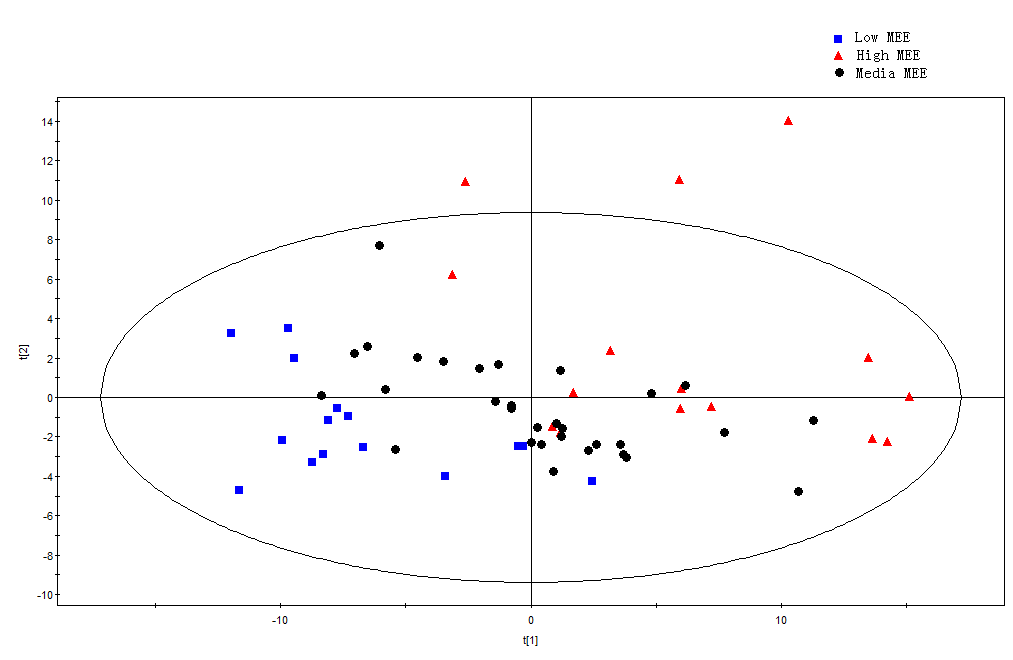


Figure S1. PLS score plot of three MEE groups

Supplement: Figure S1 — PLS score plot of three MEE groups. (DOC) [file pone.0088102.s001.doc]

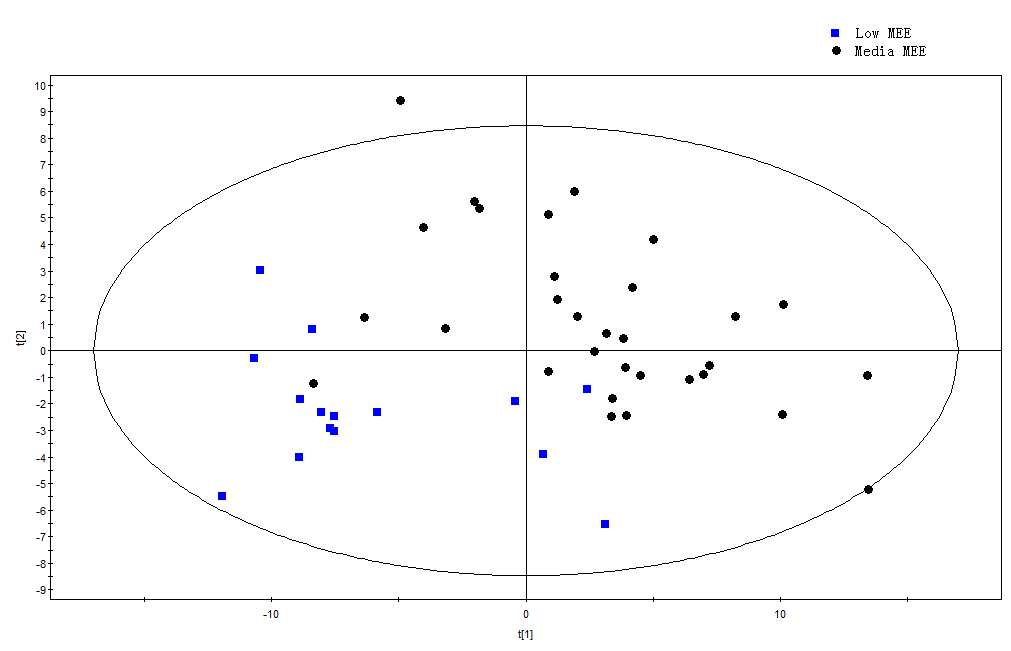


Figure S2. PLS score plot of low MEE and intermediate MEE

Supplement: Figure S2 — PLS score plot of low MEE and intermediate MEE. (DOC) [file pone.0088102.s002.doc]

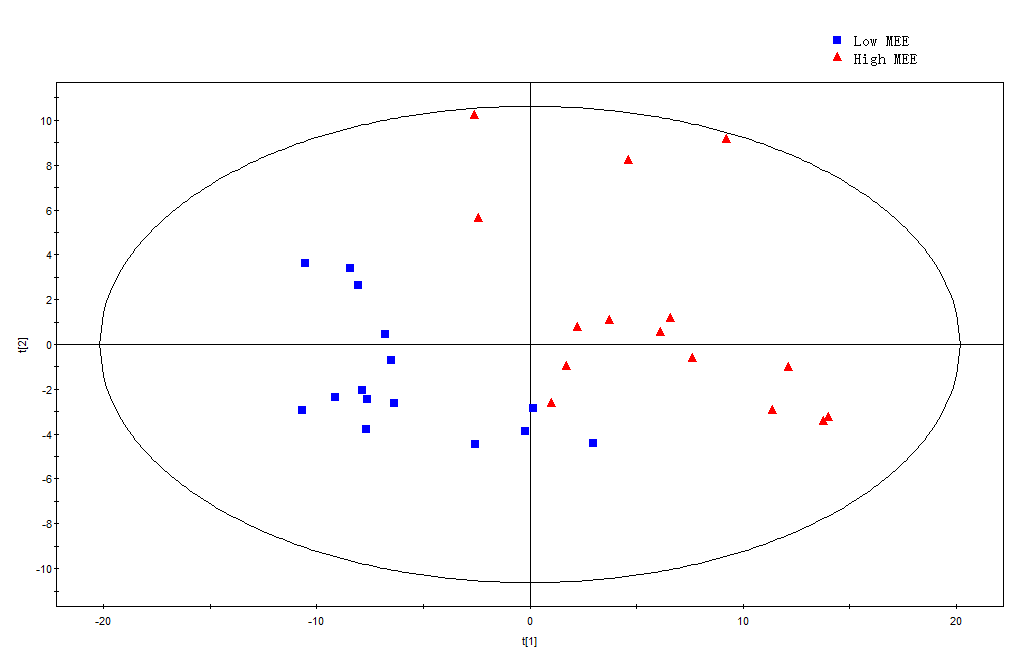


Figure S3. PLS score plot of low MEE and high MEE

Supplement: Figure S3 — PLS score plot of low MEE and high MEE. (DOC) [file pone.0088102.s003.doc]
